# Evaluation of Postoperative Deficits following Motor Cortex Tumor Resection using Small Craniotomy

**DOI:** 10.1055/s-0039-1679931

**Published:** 2019-03-04

**Authors:** Wellingson Silva Paiva, Erich Talamoni Fonoff, André Beer-Furlan, Bárbara Albuquerque Morais, Iuri Santana Neville, Rogério Bicudo Ramos-Filho, Manoel Jacobsen Teixeira

**Affiliations:** 1 Faculdade de Medicina, Hospital das Clínicas HCFMUSP, Universidade de Sao Paulo, Sao Paulo, Brazil; 2 Faculdade de Medicina, Universidade de Sao Paulo, Sao Paulo, Brazil; 3 Department of Neurological Surgery, Wexner Medical Center, The Ohio State University College of Medicine, Columbus, Ohio; 4 Instituto do Cancer do Estado de Sao Paulo ICESP, Faculdade de Medicina, Universidade de Sao Paulo, Sao Paulo, Brazil

**Keywords:** brain neoplasms, motor cortex, brain mapping, craniotomy

## Abstract

**Introduction** Surgical treatment of brain tumors in eloquent areas has always been considered a major challenge because removal-related cortical damage can cause serious functional impairment. However, few studies have investigated the association between small craniotomies and the higher risk of incidence of motor deficits and prolonged recovery time. Here, we analyzed neurologic deficits and the prognostic variables after surgery guided by navigation for motor cortex tumors under general anesthesia.

**Methods** This was a prospective study that included 47 patients with tumors in the precentral gyrus. All surgeries were performed with neuronavigation and cortical mapping, with direct electrical stimulation of the motor cortex. We evaluated the prognostic evolution of patients with pre- and postoperative Karnofsky Performance Scale using the Eastern Cooperative Oncology Group scale.

**Results** Complete resection was verified in all 18 cases of metastasis, 13 patients with glioblastoma multiforme, and 5 patients with low-grade gliomas. An analysis of the motor deficits revealed that 11 patients experienced worsening of the deficit on the first day after surgery. Only four patients developed new deficits in the immediate postoperative period, and these improved after 3 weeks. After 3 months, only two patients had deficits that were worse those experienced prior to surgery; both patients had glioblastoma multiforme.

**Conclusion** In our series, motor deficits prior to surgery were the most important factors associated with persistent postoperative deficits. Small craniotomy with navigation associated with intraoperative brain mapping allowed a safe resection and motor preservation in patients with motor cortex brain tumor.

Surgical treatment of brain tumors in eloquent areas has always been considered a major challenge because removal-related cortical damage can cause serious functional impairment. 1 2 The phenomenon of neural plasticity in cortical–subcortical circuits in response to various clinical situations and the variability among individuals with different hodological features make it difficult to establish a treatment guided solely by anatomy. 3 4 5 As such, the use of mapping by direct electrical stimulation of the cerebral cortex proposed by Penfield became the standard method to guide the brain tumor resections. 1 6

Although innovative preoperative mapping methods, such as functional transcranial magnetic stimulation and positron emission tomography, have become more widely available, direct electrical stimulation continues to be the standard method. 1 7 8 9 The challenge in surgery is to fully resect the lesion while minimizing the risk of inducing motor deficits. Usually, this mapping involves large craniotomy for cortical exposure. 10 11 12 13 However, few studies have analyzed clinical aspects, such as incidence of motor deficits, and recovery time using small craniotomies. Here, we analyzed neurologic deficits and the prognostic variables in a series of 47 patients who underwent surgery guided by navigation for motor cortex tumors under general anesthesia.

## Material and Methods

### Study Design

**Fig. 1:**
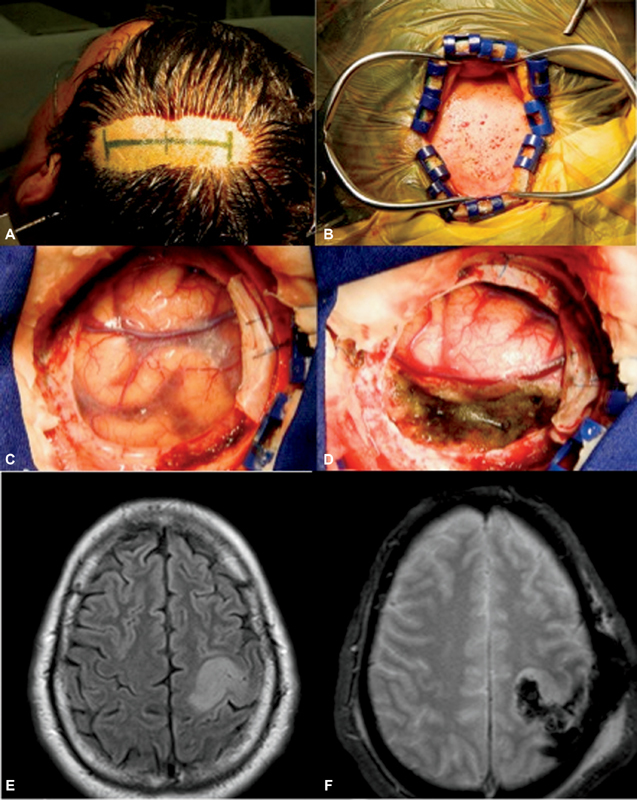
Standard approach to brain tumor in the motor cortex. A 56-year-old patient presented with seizures and low-grade glioma in the pre- and postcentral gyrus. ( **A** ) Head position and incision mark guided by navigation. ( **B** ) Incision and skull exposure. ( **C** ) Motor cortex and tumor exposure for brain mapping. ( **D** ) After resection in a patient, without postoperative deficits. ( **E** ) Preoperative magnetic resonance imaging (MRI) showing glioma in the motor area. ( **F** ) Postoperative MRI showing subtotal resection of the tumor.

We performed a prospective study that included 47 consecutive patients with tumors in the precentral gyrus who underwent surgery between July 2009 and January 2013 in the Division of Neurosurgery, University of Sao Paulo Medical School. The sample comprised patients with metastatic and primary lesions, with an average age of 45.66 ± 14.62 years, ranging from 18 to 70 years, operated using small craniotomy guided by navigation with tumor and partial motor cortex exposure ( Fig. 1 ).

Lesion proximity was defined considering tumor in or within 5 mm of the precentral gyrus distance. We excluded patients with Karnofsky Performance Scale (KPS) scores < 70, those between 18 and 70 years of age, and individuals with multiple brain lesions.

The study was approved by our institutional ethics committee (CAPPEsq [Ethics Committee for Analysis of Research Projects] of our institution) and was in accordance with the Declaration of Helsinki. All patients agreed to participate in the study and signed the informed consent form.

### Outcomes

All patients were subjected to clinical and neurologic examinations and were evaluated for the presence of motor deficit according to the British Council of Medical Research rating scale (from 0 to 5). This evaluation was performed considering the worst motor deficit in muscle groupings contralateral to the cerebral lesion. After the surgical procedure, other similar evaluations were performed on the first, second, and third postoperative day and repeated 1 and 3 months after surgery. We evaluated the prognostic evolution of patients with pre- and postoperative KPS using the Eastern Cooperative Oncology Group (ECOG) scale.

### Anesthesia

In all cases, intravenous anesthesia was performed without the administration of volatile anesthetics. No muscle-relaxing drugs were used after patients' intubation. Induction of anesthesia was achieved by propofol infusion (1–2 mg/kg) and fentanyl (5–10 mg/kg). Anesthesia was maintained by the continuous administration of propofol (75–125 mg/kg). Analgesia was achieved with fentanyl (2 mg kg ^−1^ ). In all patients, fentanyl–propofol administration was reduced 20 minutes before intraoperative cortical mapping began.

### Intraoperative Mapping

All surgeries were performed with neuronavigation (Vector vision, Brainlab, Munich, Germany) and cortical mapping with direct electrical stimulation of the motor cortex.

Skin incision and craniotomy were guided by neuronavigation, and the tumor and precentral gyrus were exposed after craniotomy as little as possible. Preoperative planning was performed with three-dimensional reconstruction of each patient's brain magnetic resonance imaging (MRI) and included a margin of 1 cm of primary motor cortex beyond the tumor's surface projection.

After skull opening, using a small craniotomy guided by navigation ( Fig. 1 ), we used a bipolar dural stimulator. The stimulation pen was placed in contact with the exposed cortical site corresponding to a particular muscle to verify the efficacy of the blocking anesthesia. The stimulation parameters were set on a case-by-case basis, with stimulation current between 1 and 10 mA, a fixed frequency of 60 Hz, and pulse durations of 100 ms using a bipolar stimulation system (Micromar, Sao Paulo, Brazil). All exposed cortical surfaces were mapped, including the region affected by the tumor, including the pre- and postcentral gyri. We started with low intensity and gradually increased the stimulation. The lowest possible stimulus that evoked a response was recorded and used for topographic evaluation. Subcortical stimulation was performed with the same settings and current intensity of cortical mapping as needed.

No correlation between the required current intensity to evoke movement and postoperative weakness was found. The current intensity varies, depending on many factors including the anesthetic condition of the patient.

### Statistical Analysis

The data were tabulated using SPSS 17.0 (SPSS Inc., Chicago, IL). The Kolmogorov–Smirnov test was applied to assess whether the data were normally distributed. All tests were performed with a significance level of 5%.

## Results

Among the 47 surgeries, 16 were performed in patients with a diagnosis of glioblastoma multiforme, 13 patients had low-grade gliomas, and 18 patients had brain metastases. Among low-grade gliomas, nine were diffuse astrocytomas and four were oligoastrocytomas. In the group of patients with metastasis, 14 patients had primary cancer of the lung, 2 cases had gastrointestinal tract tumors, and 2 had melanoma. An intraoperative brain mapping was performed in all patients, independently of the size of their craniotomy. In 11 (23.4%) patients, cortical mapping revealed that functional areas had been invaded by the tumor, and incomplete resection was performed to avoid permanent postoperative deficits ( Table 1 ).

**Table 1:** Distribution of patients according to clinical and radiological characteristics

	*N*	%
Histology		
Glioblastoma	16	34
Low-grade glioma	13	27.6
Metastasis	18	38.3
Sex		
Male	28	59.5
Female	19	40.5
First symptom		
Headache	21	44.7
Motor deficit	13	27.6
Seizure	07	14.9
Other	06	12.8
Preoperative deficit		
Yes	19	40.5
No	28	59.5
Functional activity in the tumor area		
Yes	11	23.4
No	36	76.6
Resection		
Total gross resection	36	76.6
Subtotal resection	09	19.1
Biopsy	02	4.2
Immediate postoperative deficits		
Yes	15	31.9
No	32	68.1
Late postoperative deficits		
Yes	2	4.25
No	45	95.75

Macroscopic resection was performed in 36 complete resection surgeries. Complete resection was verified in all 18 cases of metastasis, 13 patients with glioblastoma multiforme, and 5 patients with low-grade gliomas. Subtotal resection was performed in three patients with glioblastoma multiforme and six patients with low-grade glioma. In two patients with low-grade glioma, only biopsies were performed because cortical electrical stimulation revealed that almost the entire length of the tumor had invaded the motor area.

An analysis of the motor deficits revealed that 19 patients had experienced some degree of preoperative motor deficit. Of these, 11 experienced worsening of the deficit on the first day after surgery. Only four patients developed new deficits in the immediate postoperative period, and these improved after 3 weeks. After 3 months, only two patients had deficits that were worse than those experienced prior to surgery; both patients had glioblastoma multiforme with worsening of the deficit from grade IV to grade II. Among the 19 patients who had deficits, 17 initially presented deficit improvement, and the deficits persisted in 2 patients at the 3-month postoperative evaluation. In patients who had experienced deficits prior to surgery, the incidence of worsening in the immediate postoperative period (57.90% × 14.29%, *p* = 0.001) was significantly greater than in those who did not have preoperative deficits ( Table 2 ).

**Table 2:** Relationship between tumor characteristics and motor deficits in the immediate postoperative time

	% of patients with motor deficits in the immediate postoperative time	*p* -Value
Tumor diameter		
< 4 cm	22.22	0.04
> 4 cm	45	
Distance between the tumor and the motor cortex		
< 5 mm	39.20	0.22
> 5 mm	47.30	
Preoperative deficits		
Yes	57.90	0.002
No	14.29	
Intraoperative seizure		
Yes	42.80	0.71
No	40	

We verified intraoperative seizure in 7 of 47 patients during direct cortical stimulation; however, we did not find a correlation between the stimulation current and the history of epilepsy or intraoperative seizure ( Table 1 ).

Surgical wound dehiscence occurred in four patients and was corrected with sutures when the patients return to the emergency room of the institution. Bacterial meningitis in a pulmonary venous thrombosis occurred in two other patients (one with metastasis and another with glioblastoma multiforme) and was treated with anticoagulation.

Regarding tumor volume and worsening motor deficits, we found that the tumor was significantly larger in patients who experienced worsened deficits in the immediate postoperative period ( *p* = 0.04). However, when we analyzed the relationship between tumor volume and late postoperative motor deficits, we did not find a statistically significant difference ( *p* = 0.82) ( Table 2 ).

We assessed the relationship between postoperative deficit worsening and lesion proximity to the motor cortex and did not find a difference between patients with injury involving the motor cortex compared with those with lesions adjacent to the motor cortex (>5 mm from the motor cortex).

The KPS evaluation revealed improvement in a proportion of patients in the series (average preoperative KPS of 79.5 vs. average postoperative KPS of 87; *p* = 0.013 and *p* = 0.013, respectively). Using the ECOG scale to assess the preoperative scores and improvement 3 months after surgery, we also found a statistically significant improvement ( *p* = 0.006). There was no relationship between tumor volume, epilepsy, or age, and postoperative performance. However, preoperative deficit ( *p* = 0.021) and edema intensity (group without edema vs. group with edema; *p* = 0.01) were found to be associated with improvement in patient performance using KPS 3 months after surgery.

## Discussion

The current recommendation for the treatment of intracranial neoplasms is resection of the lesion to a degree that will maintain the integrity of neurologic function followed by neuroimaging and oncological treatment such as chemotherapy or radiation when appropriate. 14 Wood et al 15 performed a retrospective study and compared morbidity and mortality in patients with tumors in eloquent versus those with lesions in other areas. The relationship between the distance from the tumor to the motor area, and the incidence of functional deficits and survival was also evaluated. There was a significant association between deficits and the distance from the edge of the tumor to the motor area. A previous study reported that the prevalence of motor deficits increased linearly at ∼39% when the tumor was 1 and 2 cm from the motor area compared with distances greater than 2 cm. Moreover, a 34% increase in deficit prevalence was verified in cases where the distance was less than 1 cm. 16 In this series, the distance from the tumor to the motor cortex was not associated with KPS or ECOG scores.

Although surgery for tumors adjacent to the motor cortex has been the subject of much controversy in the literature, a growing number of studies have provided evidence for the positive impact of total resection of these tumors in the treatment of the patients. 17 18 However, proximity to eloquent areas often limits the extent of resection. In our series, we found that tumors near the motor cortex more often necessitated subtotal and partial resections. Studying specifically low-grade gliomas, Duffau et al 17 reported that 50.8 and 25.4% subtotal and total resections, respectively. In our series, gross resections were possible in 76% of the operations, subtotal resection in 20%, and in two patients, only biopsy was performed.

In another study, Kim et al 18 using large craniotomy reported that radical tumor resection was achieved in 199 procedures (64%), subtotal resection was performed in 43 (14%) patients, and a partial resection was performed in 67 (22%). Moreover, total resection was achieved in 73% of recurrent tumors, 61% of anaplastic tumors, 39% of low-grade tumors, and 95% of metastatic tumors. Total resection was verified in 112 of 181 newly diagnosed tumors (62%), 76 of 109 recurring tumors (70%), and 11 of residual tumors 19 (58%). In our study, using small craniotomy with navigation, we achieved total resection in 8 of the 9 patients with glioblastoma and in all 18 patients with metastatic cancer.

Brainer-Lima et al 19 studied 42 patients with lesions adjacent to the motor cortex who underwent craniotomy with cortical stimulation. Surgical resection was achieved in 29 (69.1%) patients. Subtotal resection was verified in eight (33.3%) patients with primary tumor and in one patient (5.6%) with a secondary tumor. Partial resection was performed in four (16.7%) patients with primary tumors. Sarmento et al 20 described a series of 42 patients with complete and subtotal surgical resection in 38 (90.4%) and 4 (9.5%) cases, respectively.

Regarding the relationship between pre- and postoperative deficits and tumor proximity, we found that preoperative motor deficits were related to the manifestation of postoperative deficits. This finding suggests that surgery to resect motor area tumor using small craniotomy does not significantly increase morbidity in these patients. In our series, 30% of cases showed neurologic worsening in the immediate postoperative period, and 4.2% of the patients exhibited some persistent deficit. However, when we evaluated patients with previous deficits, the incidence of worsening in the immediate postoperative period was significantly higher in those who did not experience preoperative deficits. Duffau et al 21 using a large craniotomy reported that 6% of patients experienced permanent motor deficit; however, they reported 78% with an immediate deficit. Keles et al 22 described a total of 60 (20.4%) patients who experienced an additional motor deficit in the postoperative period. Of these, 23 (38.3%) patients recovered in the first postoperative week, and postoperative motor deficits were resolved by the end of the first month in 12 (20%) patients. Motor deficits were found in 11 (18.3%) of 60 patients who continued to improve until the end of the third month. A total of 14 (4.8%) patients had persistent deficits after 3 months. In our study, only two patients continued to experience motor deficit at the 3-month evaluation. Among 14 patients with persistent deficits described by Keles et al, 8 had neurologic deficit (grade 4/5). Three patients presented moderate deficits (3/5), whereas severe neurologic deficits were identified in the other three. In our sample, two patients experienced worsening from grade IV to grade II.

Keles et al 22 reported that the incidence of new temporary and permanent motor deficits is related to the presence of a preoperative neurologic deficit. They performed multivariate analysis and showed that the presence of preoperative motor deficit was an independent risk factor for an additional motor deficit in the postoperative period. Patients who had motor deficits prior to surgery were more likely to develop an additional postoperative motor deficit compared with those who had preserved motor functions (25.8% compared with 16.5%). However, the presence of a preoperative motor deficit was not a predictor of developing a permanent postoperative motor deficit. In our sample of 47 patients, 19 had preoperative motor deficits; 57.9% experienced worsening of the deficit in the immediate postoperative period, whereas only 14.29% of 28 patients without prior motor deficit developed postoperative motor deficits ( *p* = 0.002).

## Conclusions

Resection of tumors in motor areas is a major challenge for neurosurgical teams. In our series, motor deficits prior to surgery were the most important factors associated with persistent postoperative deficits. Small craniotomy with navigation allowed a safe resection of the motor cortex brain tumor; however, further studies are needed to define the role of small craniotomy with navigation compared with large conventional craniotomy.
